# Clinical Profile, Etiology, and Outcomes of Patients With Eosinophilia: A Prospective Study From Western India

**DOI:** 10.7759/cureus.73406

**Published:** 2024-11-10

**Authors:** Sushrut Fuladi, Neelam Redkar, Nitin Sarate, Shifa Karatela, Alhad Mulkalwar

**Affiliations:** 1 Department of Nephrology, Lokmanya Tilak Municipal Medical College and General Hospital, Mumbai, IND; 2 Department of Medicine, Hinduhridaysamrat Balasaheb Thackeray Medical College and Dr. R. N. Cooper Municipal General Hospital, Mumbai, IND; 3 Department of Medicine, King Edward Memorial Hospital and Seth Gordhandas Sunderdas Medical College, Mumbai, IND; 4 Department of Medicine, Medical College Baroda and Sir Sayajirao General Hospital, Vadodara, IND; 5 Department of Pharmacology, Dr. D. Y. Patil Medical College, Hospital and Research Centre, Dr. D. Y. Patil Vidyapeeth (Deemed to Be University), Pune, IND

**Keywords:** allergic eosinophilia, atopy, blood hypereosinophilia, eosinophilia, ige

## Abstract

This study investigated the clinical profile, etiology, and outcomes of eosinophilia in hospitalized patients. A total of 96 patients were included, with a mean eosinophil count of 3347.66 ± 1517.39 at admission, which decreased to 1265.39 ± 549.42 at discharge. The majority of patients were males. Common presenting symptoms included anorexia, abdominal pain, fever, fatigue, breathlessness, and cough. Idiopathic eosinophilia was the most frequent diagnosis, followed by parasitic infestations and atopy. Hypertension and ischemic heart disease were common co-morbid conditions. Elevated serum IgE levels were significantly associated with the severity of eosinophilia. Patients with severe eosinophilia had longer hospital stays and higher mortality rates, with no deaths observed in the mild eosinophilia group. The findings underscore the importance of thorough diagnostic evaluation, assessment of organ involvement, and management of associated co-morbidities.

## Introduction

Blood hypereosinophilia (HE) is a common clinical finding across various medical fields. Interleukin (IL)-5, produced mainly by T cells, plays a key role in eosinophil development [1]. HE can be classified as primary, secondary, hereditary, or idiopathic. It is defined by a blood eosinophil count exceeding 1.5x10^9^/L on two occasions within a month and/or tissue involvement [2]. Severe eosinophilia or accompanying symptoms like fever, rash, or organ damage prompt further investigation to identify underlying causes, including allergies, infections, or blood disorders [3].

Even after identifying the cause of eosinophilia, patients need ongoing monitoring for potential organ damage caused by the condition or the underlying disease. Eosinophilia presents an interesting challenge due to its often vague presentation: there are typically no definitive symptoms, and in many cases, no clear cause is identified. Most patients with eosinophilia are asymptomatic, with the condition usually being detected incidentally during routine hematological tests.

Most patients with eosinophilia are managed on an outpatient basis, though some require hospitalization. Currently, there is no established protocol for investigating these patients. In this prospective study, we aimed to assess the clinical profiles and associated etiologies of patients hospitalized with eosinophilia to help develop guidelines for the work-up and diagnosis of such cases.

## Materials and methods

A prospective observational study aimed to assess the clinical profiles and outcomes of patients with eosinophilia was conducted over 18 months from July 2016 to December 2017 in the general medicine ward of a tertiary care hospital with approval from the Institutional Ethics Committee (approval number: EC/658/16). A total of 96 participants were included based on admission, all of whom were over 12 years old and had an absolute eosinophil count (AEC) greater than 500/µL. Patients who had already received treatment for eosinophilia and pregnant patients were excluded.

Upon enrollment, detailed histories and physical examinations were conducted. Routine hematological and biochemical tests like complete blood count test, erythrocyte sedimentation rate (ESR), blood glucose levels, serum creatinine, lactate dehydrogenase (LDH), albumin, and serum glutamic pyruvic transaminase (SGPT) levels, and additional diagnostic workups performed by the treating physicians to determine the cause of eosinophilia were noted.

Participants were then categorized into three groups based on their AEC, following Marc E. Rothenberg's 1998 classification: mild (500-1500/µL), moderate (1500-5000/µL), and severe (more than 5000/µL) [4]. The patient's clinical course, from admission to final outcome, including diagnosis or death, was tracked.

Data was entered into MS Excel 2021 (Microsoft® Corp., Redmond, WA, USA). Data analysis was conducted using SPSS Statistics for Windows, Version 17.0. Chicago: SPSS Inc. Quantitative data is expressed as mean ± standard deviation (SD) and qualitative data as percentages. Chi-squared test was used to assess relationships between categorical variables, with a two-tailed p-value of less than 0.05 considered statistically significant.

The study outcome was to assess the distribution of eosinophilic patients in relation to age, gender, and severity, identify the frequency of various causes of eosinophilia, assess the clinical and hematological profiles of these patients, and evaluate outcomes in terms of diagnosis and mortality.

## Results

In this study, it was observed that the majority of patients belonged to the 21-30 years age group, n = 25 (26.04%), followed by those in the 31-40 years age group, n = 19 (19.79%). Additionally, a greater proportion of the patients, 60.42%, were male. Regarding the severity of eosinophilia, most patients exhibited moderate eosinophilia, followed by mild eosinophilia. It was also noted that mild eosinophilia was more common among males and in the 21-40 years age range. However, no significant association was found between the severity of eosinophilia and either age (X² (6, n = 96) = 3.43, p = 0.75) or gender (X² (2, n = 96) = 0.25, p = 0.88). This indicates that while certain demographic trends were observed, they did not show a statistically significant correlation with the severity of eosinophilia (Table 1).

**Table 1:** Age, sex, and severity-wise distribution of study participants

Age, n (%)	Mild, n (%)	Moderate, n (%)	Severe, n (%)
12-20 years, n = 18 (18.75%)	9 (50)	6 (33.3)	3 (16.7)
Male, n = 10 (55.6%)	5 (50)	3 (30)	2 (20)
Female, n = 8 (44.4%)	4 (50)	3 (37.5)	1 (12.5)
21-40 years, n = 44 (45.8%)	13 (29.5)	23 (52.3)	8 (18.2)
Male, n = 29 (65.9%)	10 (34.5)	14 (48.3)	5 (17.2)
Female, n = 15 (34.1%)	3 (20)	9 (60)	3 (20)
41-60 years, n = 22 (22.9%)	6 (27.3)	11 (50)	5 (22.7)
Male, n = 13 (59.1%)	3 (23.1)	7 (53.8)	3 (23.1)
Female, n = 9 (50.1%)	3 (33.4)	4 (44.5)	2 (22.2)
>60 years, n = 12 (12.5%)	4 (33.3)	5 (41.7)	3 (25)
Male, n = 6 (50%)	2 (33.3)	2 (33.3)	2 (33.3)
Female, n = 6 (50%)	2 (33.3)	3 (50)	1 (16.7)
Total, n = 96 (100%)	32 (33.3)	45 (46.9)	19 (19.8)
Total male, n = 58 (60.4%)	20 (34.5)	26 (44.8)	12 (20.7)
Total female, n = 38 (39.6%)	12 (31.6)	19 (50)	7 (18.4)

The data revealed that the majority of patients, 53 (55.21%), were hospitalized for the first time, while 29 (30.21%) had a second hospitalization due to eosinophilia, and 14 (14.58%) had been admitted more than twice. The most frequently reported symptoms included anorexia, abdominal pain, fever, fatigue, breathlessness, and cough (Table 2).

**Table 2:** Distribution of presenting symptoms according to the severity of eosinophilia

Symptom, n (%)	Mild, n = 32 (33.3%)	Moderate, n = 45 (46.9%)	Severe, n = 19 (19.8%)
Anorexia, n = 38 (39.58%)	9 (23.68)	12 (31.58)	17 (44.74)
Arthralgia, n = 17 (17.71%)	5 (29.41)	8 (47.06)	4 (23.53)
Breathlessness, n = 29 (30.21%)	6 (20.69)	14 (48.28)	9 (31.03)
Chest pain, n = 8 (8.33%)	2 (25)	4 (50)	2 (25)
Cough, n = 21 (21.88%)	9 (42.86)	7 (33.33)	5 (23.81)
Diarrhea, n = 10 (10.42%)	5 (50)	3 (30)	2 (20)
Fatigue, n = 29 (30.21%)	12 (41.38)	9 (31.03)	8 (27.59)
Fever, n = 31 (32.29%)	17 (54.84)	8 (25.81)	6 (19.35)
Myalgia, n = 12 (12.5%)	3 (25)	7 (58.33)	2 (16.67)
Abdominal pain, n = 36 (37.5%)	8 (22.22)	16 (44.44)	12 (33.33)
Palpitation, n = 6 (6.25%)	0 (0)	2 (33.33)	4 (66.67)
Rash/urticaria, n = 21 (21.88%)	9 (42.86)	7 (33.33)	5 (23.81)
Rhinitis/cough, n = 15 (15.63%)	8 (53.33)	4 (26.67)	3 (20)
Syncope/giddiness, n = 5 (5.21%)	0 (0.00)	3 (60.00)	2 (40.00)
Urinary, n = 6 (6.25%)	1 (16.67)	2 (33.33)	3 (50.00)
Weakness, n = 22 (22.92%)	9 (40.91)	7 (31.82)	6 (27.27)
Weight loss, n =12 (12.50%)	2 (16.67)	4 (33.33)	6 (50.00)
Worms in stool, n = 18 (18.75%)	4 (22.22)	8 (44.44)	6 (33.33)
Total, n = 96 (100%)	32 (33.3)	45 (46.9)	19 (19.8)

The majority of patients (35.42%) were found to have idiopathic eosinophilia. The mean eosinophil count was 3393 ± 1026 in idiopathic eosinophilia cases. The highest mean eosinophil count, however, was observed in patients with hematological malignancies, reaching 7139 ± 535 (Table 3).

**Table 3:** Distribution according to etiology with mean eosinophil counts

Etiology	No. of patients n = 96 (%)	Mean eosinophil blood count (cell/μL) (mean ± SD)
Idiopathic	34 (35.42)	3393 ± 1026
Parasitic	18 (18.75)	3723 ± 1440
Atopy	14 (14.58)	2064 ± 1596
Hematological malignancy	6 (6.25)	7139 ± 535
Filariasis	4 (4.17)	5315 ± 848
Hypereosinophilic syndrome	4 (4.17)	5754 ± 792
Other infection	4 (4.17)	3762 ± 1341
Eosinophilic pneumonia	3 (3.13)	5497 ± 814
Churg Strauss syndrome	3 (3.13)	5581 ± 3748
Skin disease	3 (3.13)	1390 ± 818
Allergic Bronchopulmonary pneumonia (ABPA)	2 (2.08)	5513 ± 745
Drug-induced	1 (1.04)	2141 ± 725

Hypertension (30.21%) and ischemic heart disease (29.17%) were the most common co-morbid conditions in eosinophilic patients (Table 4).

**Table 4:** Distribution according to co-morbid conditions

Comorbidity	No. of patients, n (%)
Hypertension	29 (30.21)
Ischemic heart disease	28 (29.17)
Anemia	18 (18.75)
Obstructive lung disease	14 (14.58)
Renal failure	12 (12.50)
Neoplasia	8 (8.33)
Diabetic mellitus	8 (8.33)
Cerebrovascular disease	6 (6.25)
Thromboembolism	6 (6.25)
Hypothyroidism	4 (4.17)
Peripheral vascular disease	2 (2.08)

Table 5 presents the mean distribution of laboratory investigations categorized by the severity of eosinophilia.

**Table 5:** Routine laboratory investigations and their distribution according to the severity of eosinophilia WBC: white blood cell; LDH: lactate dehydrogenase; SGPT: serum glutamic pyruvic transaminase; ESR: erythrocyte sedimentation rate

Test name	No. of patients tested n=96 (%)	Mean ± SD	Mild (mean ± SD)	Moderate (mean ± SD)	Severe (mean ± SD)
Hemoglobin (g/dL)	96 (100)	11.13 ± 1.23	11.36 ±1.54	10.65 ± 1.24	10.12 ± 1.38
Platelets (10^9^ cells/L)	96 (100)	256.32 ± 118.67	287.47 ± 117.54	258.36 ± 119.38	255.3 ± 122.67
WBC (cells/μL)	96 (100)	13253 ± 9653	14325 ± 8765	13386 ± 9865	13178 ± 9387
Glucose (mg/dL)	85 (88.5)	109.32 ± 32.7	112.38 ± 32.65	111.54 ± 34.34	108.47 ± 31.24
Creatinine (mg/dL)	82 (85.4)	1.69 ± 0.9	1.24 ± 0.9	1.49 ± 1.03	1.89 ± 1.11
LDH (U/L)	75 (78.1)	298.72 ± 94.27	274.76 ± 97.45	286.38 ± 96.35	302.78 ± 99.28
Albumin (g/dL)	75 (78.1)	45.2 ± 8.42	42.23 ± 7.97	45.3 ± 8.34	47.2 ± 9.12
SGPT (U/mL)	67 (69.8)	36.26 ± 19.21	34.32 ± 21.23	36.32 ± 22.23	38.43 ± 25.34
ESR (mm/hr)	67 (69.8)	57.42 ± 25.29	52.83 ± 25.76	55.38 ± 27.43	59.38 ± 22.99

Anti-neutrophilic cytoplasmic antibodies were tested in 44 patients, with positive results in three cases. The rheumatic factor was positive in 2 out of 40 cases (Table 6).

**Table 6:** Findings of laboratory investigation done by treating physician

Investigation	Number tested, n (%)	Positive findings, n (%)
Specific IgE tests	96 (100)	54 (56.2)
ANCA (anti-neutrophilic cytoplasmic Ab)	44 (45.8)	3 (6.8)
Rheumatic factor	40 (41.7)	2 (5)
Other autoantibodies	40 (41.7)	1 (2.5)
Antinuclear antibodies	34 (35.4)	1 (2.9)
Chest CT	33 (34.4)	12 (36.4)
Tuberculin test	28 (29.2)	5 (17.8)
Echocardiograph	21 (21.9)	9 (42.8)
Bone marrow biopsy	21 (21.9)	12 (57.1)
Pulmonary function test	16 (16.7)	7 (43.7)
Allergy tests	11 (11.5)	7 (63.6)
Head CT	9 (9.4)	2 (22.2)
Worms in stool	25 (26.04)	18 (72)
Sputum	12 (12.5)	4 (33.3)

Most patients with mild eosinophilia had symptoms affecting only one system, whereas those with moderate and severe eosinophilia exhibited symptoms across two or more systems, with this association being statistically significant (p < 0.001). Respiratory symptoms were more frequently observed in patients with mild and moderate eosinophilia, while gastrointestinal symptoms were more common in patients with moderate eosinophilia. However, this association was not statistically significant (p = 0.67). Elevated serum IgE levels were found in 54 patients, and the association between eosinophilia and high serum IgE levels was statistically significant (p = 0.005). The mean AEC decreased from 3347.66 ± 1517.33 at admission to 1265.39 ± 549.42 at discharge. Most patients were hospitalized for less than seven days (55.21%), with 20.83% staying 8-14 days and 15.63% staying 15-21 days. The association between eosinophilia severity and hospital stay duration was statistically significant (p < 0.001). Out of 96 patients, 88 (91.67%) survived, and eight (8.33%) died. Among the 62 diagnosed patients, 56 survived and six died. Of the 34 idiopathic eosinophilia patients, 32 survived and two died. Of the eight deaths, three were in patients with moderate eosinophilia and five in those with severe eosinophilia. No deaths occurred among patients with mild eosinophilia. The differences in mortality rates by eosinophilia severity were statistically significant, indicating a significant association between eosinophilia severity and patient outcomes (p = 0.004) (Table 7).

**Table 7:** Association of systemic involvement, serum IgE levels, hospital stay, and outcome with severity of eosinophilia Statistically significant p-values are in bold.

Parameter	Severity of eosinophilia, n (%)			Chi-square test
	Mild	Moderate	Severe	
Number of systems, n (%)				
One system, n = 47 (48.9%)	24 (51.1)	18 (38.3)	5 (10.6)	X^2^ (2, n = 96) = 14.03, p < 0.001
≥2 Two Systems, n = 49 (51.1%)	8 (16.3)	27 (55.1)	14 (28.6)	
Type of system, n (%)				
Respiratory system, n = 57 (59.4%)	21 (36.8)	25 (43.9)	11 (19.3)	X^2^ (2, n = 96) = 0.807, p = 0.67
Gastrointestinal system, n = 39 (40.6%)	11 (28.2)	20 (51.3)	8 (20.5)	
Serum IgE, n (%)				
Raised, n = 54 (56.2%)	12 (37.5)	26 (57.8)	16 (84.2)	X^2^ (2, n = 96) = 10.6, p = 0.005
Normal, n = 42 (43.8%)	20 (62.5)	19 (42.2)	3 (15.8)	
Days hospitalized, n (%)				
≤7 days, n = 53 (55.21%)	23 (43.4)	28 (52.8)	2 (3.8)	X^2^ (8, n = 96) = 37.1, p < 0.001
8-14 days, n = 20 (20.83%)	7 (35)	10 (50)	3 (15)	
15-21 days, n = 15 (15.63%)	2 (13.3)	5 (33.3)	8 (53.3)	
22-28 days, n = 6 (6.25%)	0 (0)	2 (33.3)	4 (66.7)	
>28 days, n = 2 (2.08%)	0 (0)	0 (0)	2 (100)	
Outcome, n (%)				
Survived, n = 88 (91.67%)	32 (100)	42 (93.3)	14 (73.7)	X^2^ (2, n = 96) = 11.1, p = 0.004
Death, n = 8 (8.33%)	0 (0)	3 (6.7)	5 (26.3)	
Total, n = 96 (100%)	32 (33.3)	45 (46.9)	19 (19.8)	

## Discussion

Eosinophilia is a complex condition that requires a detailed clinical evaluation, beginning with a thorough history and physical examination. A travel history can reveal potential exposure to endemic infections such as helminthic diseases or coccidioidomycosis. Medication and dietary histories are crucial to assess allergic reactions, and symptoms related to lymphoma or adrenal insufficiency, including conditions like Addison's disease, should be investigated. Physical exams must be comprehensive, as eosinophilia can affect multiple organs, including the skin, lungs, heart, and nervous system. Laboratory investigations start with a complete blood count (CBC) with differential, and further tests like cerebrospinal fluid (CSF) analysis, urine sediment, stool studies to detect parasitic infection, and bone marrow biopsy to detect bone marrow disorders may be needed depending on suspected causes. Imaging, including CT scans, and echocardiography, can help identify organ involvement. Treatment of eosinophilia largely depends on the underlying cause, with corticosteroids commonly used for allergic and connective tissue disorders, while infections should be ruled out to prevent complications. In cases of primary eosinophilia, treatment may not be necessary unless organ involvement occurs, in which case options like corticosteroids, interferon-alpha, or newer agents such as imatinib may be utilized. Prognosis varies based on the underlying condition, degree of organ involvement, and treatment responsiveness.

The majority of patients were in the 21-30 age group (26.04%), followed by the 31-40 age group (19.79%). Similar findings have been reported by Sade et al. and Makkar et al. [3,5]. The majority of patients were male (60.42%), with a male-to-female ratio of 1.53:1. This male predominance was also observed by Sade et al. [3]. In contrast, Makkar et al. reported a female predominance, with a male-to-female ratio of 2:3 in their study [5].

Regarding the severity of eosinophilia, most patients in our study had moderate eosinophilia (46.88%), followed by mild eosinophilia (33.33%). Sade et al. found mild eosinophilia in 33% of patients, moderate in 48%, and severe in 19% [3]. Similarly, Makkar et al. reported 52% of patients with mild eosinophilia, 34% with moderate, and 14% with severe eosinophilia [5]. Helbig et al. also observed similar findings [6].

Most patients with mild eosinophilia in our study were male and aged 21-40 years. Additionally, the majority (55.21%) were hospitalized for the first time, while 30.21% had a second hospitalization due to eosinophilia. These findings are consistent with the study by Sade et al., where half of the patients were hospitalized once, and 29% were hospitalized twice, making our results comparable to this recent study [3].

The most common presenting symptoms in our study were anorexia (39.58%), abdominal pain (37.50%), fever (32.29%), fatigue (30.21%), breathlessness (30.21%), and cough (21.88%). Sade et al. reported that 20% of patients presented with constitutional systemic symptoms such as weakness, fatigue, fever, and weight loss, while 18% had cardiovascular symptoms, 12% had cutaneous symptoms, and 10% had respiratory symptoms [3]. Makkar et al. similarly observed that anorexia (40%), abdominal pain (38%), fever (32%), breathlessness (30%), cough (22%), arthralgia (18%), and myalgia (12%) were common presenting symptoms [5].

The majority of patients with mild eosinophilia in our study exhibited symptoms related to a single system, whereas those with moderate and severe eosinophilia had involvement of two or more systems. This difference was statistically significant, suggesting that severe eosinophilia is more likely to be associated with multi-system involvement. Sade et al. similarly observed that most patients had symptoms related to only one system, but those with severe eosinophilia often experienced symptoms affecting two or more systems [3]. Respiratory symptoms were most commonly associated with mild and moderate eosinophilia, while gastrointestinal symptoms were more frequent in patients with moderate eosinophilia. However, this difference was not statistically significant.

The final diagnosis in this study was determined based on clinical findings and laboratory data obtained during hospitalization. In 35.42% of cases, eosinophilia could not be linked to any identifiable disease. Despite comprehensive diagnostic evaluations, including immunological assays and imaging, all results were within normal limits, and the criteria for diagnosing hypereosinophilic syndrome (HES) were not met. Therefore, these cases were classified as eosinophilia of unknown etiology. Parasitic infestation accounted for 18.75% of cases. Atopy was present in 14.58%, and hematological malignancy caused eosinophilia in 6.25% of cases. Eosinophilia is often linked to various cancers, particularly hematologic malignancies like Hodgkin's disease and lymphomas, but can also occur in cancers of the colon, cervix, lung, breast, and ovary. Its presence does not appear to affect prognosis, and the role of eosinophils in cancer remains unclear [7,8].

In Brigden and Graydon's study, 36% of patients had idiopathic eosinophilia [9]. Sade et al. reported that asthma or other atopic diseases (e.g., hay fever) were responsible for eosinophilia in 13% of cases, allergic drug reactions in 6%, eosinophilic pneumonia in 10%, neoplastic diseases in 10%, idiopathic hypereosinophilic syndrome (IHS) in 8%, Churg-Strauss Syndrome (CSS) in 4%, infections in 10%, allergic fungal disease in 2%, and skin diseases in 3% [3]. Lombardi and Passalacqua's study found that 80% of cases were attributed to atopic diseases, followed by parasitic infestations (8.2%), hematological malignancies (2.4%), solid tumors (1.9%), gastrointestinal disorders (1.6%), and skin diseases (2.1%) [10]. Makkar et al. observed that no etiological diagnosis was found in 70% of patients, with parasitism (16%) being more common than allergic rhinitis and bronchial asthma (6%) [5]. Additionally, two patients had tuberculosis, one had viral hepatitis, and one had patchy-edematous eosinophilic dermatitis. Similar findings were reported by Rothenberg [4] and Newman [11].

Eosinophilia is linked to a wide range of diseases, making its etiology challenging to pinpoint and often requiring extensive imaging and laboratory tests [3]. The evaluation of eosinophilia is guided by its severity, associated symptoms, and initial diagnostic results, aiming to rule out treatable conditions like parasitic infections, drug allergies, or neoplasia. Even benign causes, such as allergic reactions, necessitate assessment for potential organ damage. The extent of evaluation needed remains debated [12].

In our study, the mean eosinophil count was 3393 ± 1026 in idiopathic eosinophilia, 3723 ± 1440 in parasitic eosinophilia, and 2064 ± 1596 in atopic cases. Hypertension and ischemic heart disease were the most common co-morbid conditions, which aligns with findings from Sade et al. [3].

Serum IgE levels were elevated in 54 patients, with the highest levels observed in those with moderate eosinophilia (48.15%). The association between eosinophilia severity and elevated IgE was statistically significant, consistent with findings from Makkar et al. [5].

Blood cell counts decreased with increasing severity. Overall, mean creatinine and ESR levels were elevated, and levels of creatinine, LDH, albumin, SGPT, and ESR increased progressively with the severity of the eosinophilia. Sade et al. reported similar findings with slightly higher WBC counts and normal platelet counts, but elevated creatinine levels [3]. Makkar et al. noted normocellular normochromic anemia in most patients [5]. Stool tests were positive for ova and cysts in 16% of patients, bone marrow was generally normocellular, and pulmonary function tests mostly showed a restrictive pattern. Mild anemia, leukocytosis, and elevated sedimentation rates indicated systemic involvement and inflammation.

Several studies have noted an association between eosinophilia and renal failure or hemodialysis, though the exact nature of this relationship remains unclear. Eosinophils might contribute to renal failure through direct toxicity or by causing damage to blood vessels, such as through vasculitis, immune-mediated thromboembolic events, or cholesterol emboli. Alternatively, eosinophilia and renal disease may represent two separate manifestations of an underlying condition [13].

During hospitalization, various investigations were conducted based on the differential diagnosis suggested by the patient's history, physical examination, and initial tests. Anti-neutrophilic cytoplasmic antibodies were positive in 2 out of 44 patients, and the rheumatic factor was positive in 2 out of 40 cases. Chest CT was positive in 12 out of 33 cases, and bone marrow biopsy was positive in 12 out of 21 cases.

While not all positive tests were directly related to eosinophilia, they highlight the importance of comprehensive testing and screening for potential organ damage. Chest CT and echocardiography proved valuable, supporting recommendations for cardiac and pulmonary assessments in eosinophilia patients, even if asymptomatic [11]. Pulmonary function tests, despite their high diagnostic value, were under-utilized, with 82% showing significant findings. Given that the lungs are frequently affected in eosinophilic syndromes, the importance of these tests should be emphasized. However, the study's findings are descriptive and may not apply universally; the specific use of tests may have been driven by individual symptoms. The results are consistent with findings from Sade et al. [3].

The mean AEC at admission was 3347.66 ± 1517.39, decreasing to 1265.39 ± 549.42 at discharge. Most patients were hospitalized for less than seven days (55.21%), followed by 8-14 days (20.83%) and 15-21 days (15.63%). The duration of hospital stay was statistically significant, with mild eosinophilia patients having shorter stays compared to those with moderate and severe eosinophilia. Similar findings were reported by Makkar et al. [5].

Out of 96 patients, 88 (91.67%) were discharged, and eight (8.33%) died. A diagnosis was confirmed in 62 (64.58%) cases, while 34 (35.41%) were idiopathic. Of the eight deaths, three were among patients with moderate eosinophilia and five with severe eosinophilia, with no deaths reported in patients with mild eosinophilia. The difference in mortality rates was statistically significant.

## Conclusions

Eosinophilia was predominantly observed in young male patients. The most common presenting symptoms included constitutional symptoms, along with respiratory and gastrointestinal issues. The majority of patients had idiopathic eosinophilia, followed by parasitic infestations and atopy. Elevated serum IgE levels were associated with the severity of eosinophilia. Patients with severe and moderate eosinophilia had longer hospital stays compared to those with mild eosinophilia. Of the eight deaths, three were in patients with moderate eosinophilia and five in those with severe eosinophilia, with no mortality observed among patients with mild eosinophilia.
